# Host-Parasitoid Dynamics and the Success of Biological Control When Parasitoids Are Prone to Allee Effects

**DOI:** 10.1371/journal.pone.0076768

**Published:** 2013-10-07

**Authors:** Anaïs Bompard, Isabelle Amat, Xavier Fauvergue, Thierry Spataro

**Affiliations:** 1 CNRS - Université Pierre et Marie Curie - ENS, UMR 7625 Ecologie et Evolution, Paris, France; 2 INRA, USC 2031 Ecologie des Populations et communautés, Paris, France; 3 Université de Lyon - Université Lyon 1 - CNRS, UMR 5558 Laboratoire Biométrie et Biologie Evolutive, Villeurbanne, France; 4 INRA - CNRS - Université Nice Sophia Antipolis, UMR 1355 - 7254 Institut Sophia Agrobiotech, Sophia Antipolis, France; 5 AgroParisTech, Paris, France; University of California, Berkeley, United States of America

## Abstract

In sexual organisms, low population density can result in mating failures and subsequently yields a low population growth rate and high chance of extinction. For species that are in tight interaction, as in host-parasitoid systems, population dynamics are primarily constrained by demographic interdependences, so that mating failures may have much more intricate consequences. Our main objective is to study the demographic consequences of parasitoid mating failures at low density and its consequences on the success of biological control. For this, we developed a deterministic host-parasitoid model with a mate-finding Allee effect, allowing to tackle interactions between the Allee effect and key determinants of host-parasitoid demography such as the distribution of parasitoid attacks and host competition. Our study shows that parasitoid mating failures at low density result in an extinction threshold and increase the domain of parasitoid deterministic extinction. When proned to mate finding difficulties, parasitoids with cyclic dynamics or low searching efficiency go extinct; parasitoids with high searching efficiency may either persist or go extinct, depending on host intraspecific competition. We show that parasitoids suitable as biocontrol agents for their ability to reduce host populations are particularly likely to suffer from mate-finding Allee effects. This study highlights novel perspectives for understanding of the dynamics observed in natural host-parasitoid systems and improving the success of parasitoid introductions.

## Introduction

Since the pioneering work of Nicholson and Bailey [1], many theoretical ecologists have been interested in modeling the tight interactions that occur between insect parasitoids and their hosts [2], [3]. The importance of parasitoids for pest control programs undoubtedly accounts for this interest. Because parasitoids are more specific than predators and sometimes efficient enough to cause up to 95% reduction of the host population, they have been extensively deployed as biological control agents [4]. However parasitoids released into the field often fail to establish [5], [6]. The most pervasive determinent of establishment failure is initial population size [7], [8], so that the Allee effect has been hypothesized as a likely cause of these introduction failures [7], [8], [9], [10], [11].

Demographic Allee effects are defined as decreasing *per capita* population growth rates when abundance declines. In response to “strong” Allee effects, the population growth rate becomes negative below a critical density *i.e.* the population goes extinct if its abundance falls below this threshold [12], [13], [14]. Demographic Allee effects occur in most taxa [15], and play key roles in conservation biology, invasion biology, and biological control [9], [16], [17], [18], [19]. Demographic Allee effects are underpinned by component Allee effects, namely biological traits or trophic interactions that make individual fitness positively dependent on population size [17]. The most common and most often studied component Allee effect is the mate-finding Allee effect, which describes the difficulty that males and females experience in locating each other, and the consequent decline in reproduction at low population densities. Several theoretical studies have investigated how mating difficulties may translate into demographic effects [19], [20]. Although firm evidence of causal relations between mate-finding Allee effects and establishment success in insect populations is still relatively rare [11], mating failure at low density is often considered to be an important cause of demographic Allee effects in invading species [21], [22], [23], [24], [25].

Parasitoids, in particular those introduced as biocontrol agents, are likely to experience low population densities, either because interactions with their hosts lead to cyclic dynamics, or as a consequence of external factors such as the introduction of small number of individuals in classical biological control, harvesting, crop rotation or chemical pest control [26], [27], [28]. Consequently, mate-finding difficulties may arise in parasitoid populations (the effect of number released is analyzed by Hopper and Roush [8]). Demographic Allee effects have been observed in field studies of introduced parasitoids [29], [30], but could not be linked to mate-finding difficulties in particular [29], [31].

The demographic consequences of a mating failures may be affected by other forms of density dependence in the population, such as those generated by intraspecific competition or by interactions between different trophic levels within a community [14]. The interaction between the host and the parasitoid is particularly stringent; therefore, the dynamics of host-parasitoid systems strongly depend on the characteristics of both hosts and parasitoids populations - such as intraspecific competition within the host population, the searching efficiency of the parasitoid, and the spatial distribution of parasitoid attacks [32], [33]. These biological characteristics should, in turn, combine with component Allee effects to produce idiosyncratic patterns in host-parasitoid population dynamics.

To date, Allee effects in host-parasitoid or host-parasite interactions have mainly been studied by assuming Allee effects on the host population, by considering either mate-finding [34] or demographic Allee effects [35], [36]. These have revealed the existence of a critical host density below which the parasitoid or parasite goes extinct [35], [36], but also a reduction of the chaotic domain of the dynamics [34]. Whether and how a mate-finding Allee effect in parasitoids impacts on the extinction propensity of the system remains to be investigated.

About 75% of the parasitoid species belong to the order Hymenoptera and are therefore haplodiploid (*i.e.* the males are haploid and females diploid [37], [38]). This genetic feature is important in the context of mate-finding Allee effects because it allows females to reproduce even if they do not mate: virgin females can produce males parthenogenetically by laying unfertilized eggs. Consequently, whereas a mate-finding Allee effect decreases the mean fecundity in diploid species, it produces a sex ratio shift toward males in haplodiploid ones (assuming that the fecundities of mated and unmated females do not differ [39], [40], [41], [42], [43]). Hence, it is generally accepted that haplodiploidy prevents or alleviates the demographic consequences of mate-finding Allee effects for free-living organisms [8], [44]. However, in host-parasitoid systems this assumption may not be valid because variable or density-dependent sex ratios also affect the dynamics of these systems [45], [46], [47].

In this study, we use mathematical models to tackle the dynamics of host-parasitoid systems when the parasitoid experiences mate-finding difficulties at low density. (1) We focus on the extinction propensity in relation to increasing difficulties in mating. (2) We explore the dynamics of parasitoid populations that appear persistent, and look at how the characteristics of the host (in various types of intraspecific competition) and those of the parasitoid (in various distributions of attacks) combine with mate-finding Allee effects to impact the system dynamics. (3) Finally, we adopt a more applied perspective by documenting the consequences of the mate-finding Allee effect on the minimum number of parasitoids insuring establishment and on the remanent host density.

## Materials and Methods

Following most theoretical approaches concerning demographic consequences of the Allee effect [12], [13], [19], [44], [48] we used deterministic non-spatial models. Both demographic and environemental stochasticities are known to affect the risk of extinction of a population. However, our aim is not to estimate precisely the extinction probability of a given population but rather to capture the general features of host-parasitoid systems when parasitoids are prone to an Allee effect. In this perspective, stochastic processes are probably not liable to modify significantly our qualitative conclusions. Spatial components were implicitely included in our model through functions describing the distribution of parasitoid attacks and the proportion of unmated females.

We started out from a classical discrete-time host-parasitoid framework with a host population, *N*, and a parasitoid population, *P*. At each generation, a proportion *f*(*P_t_*) of hosts escape parasitism depending on parasitoid density. The host finite rate of increase is 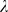 and its probability of surviving intraspecific competition is given by *g*(*N_t_*) (all parameters, their interpretation and the values used in this article can be found in Table 1). We assumed that parasitized and unparasitized hosts are equally likely to survive intraspecific competition, a situation that arises either if intraspecific competition precedes parasitism or if parasitism does not affect the competitive interactions among hosts. This gives us: 
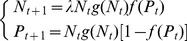
(1)


**Table 1:** Biological interpretation and numeric values for the parameters used in model simulations.

Symbol	Parameter	Values
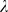	Host finite rate of increase	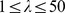
*a*	Parasitoid searching efficiency	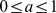
*K*	Environment carrying capacity for hosts	*K* = 500
		*b* = 0.8 (under-compensated)
*b*	Host intraspecific competition	*b* = 1.5 (mild compensation)
		*b* = 3.2 (over-compensated)
*s*_0_	Proportion of males among parasitoid offspring	*s_0_* = 0.5
		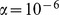 (no Allee effect)
*α*	Intensity of the mate-finding Allee effect	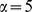 (mild Allee effect)
		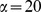 (strong Allee effect)
*k*	Distribution of parasitoid attacks	k = 0.8 (aggregated attacks)
		k = 10 (random attacks)

Males (*M*) and females (*F*) were distinguished within the parasitoid population in order to model a mate-finding Allee effect. We assumed females to produce offspring with a fixed fertilization rate 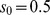 (in haplodiploids, fertilized eggs will develop into females whereas unfertilized eggs develop into males).

A consequence of the mate-finding Allee effect is that a proportion of virgin females *p(M_t_)* occurs in the population as a decreasing function of male density:



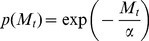
(2)
[12]where 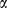 describes the intensity of the mate-finding Allee effect: for a given male density, the greater 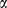, the greater the proportion of virgin females. If 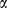 approaches 0 (no Allee effect), then *p(M_t_)* approaches 0 as well.

Because we considered an haplodiploid reproductive system, both mated and virgin females attack the hosts; we assumed that virgin females attacks the host at the same rate and have the same fecundity as mated females, but produce only male offspring. The model becomes: 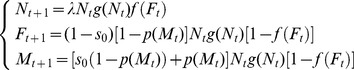
(3)


Without Allee effects (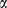 approaches 0), this model converges to the one-sex model (equation 1), which could be solved analytically (see Text S1 and Figure S1).

### Probability of escaping parasitism

To describe the probability that a host escapes from parasitism, we chose a function that allowed us to consider various possible distributions of parasitoid attacks, which is known to affect the stability of the host-parasitoid system [49]. *f* is given by the zeroth term of the negative binomial distribution: 
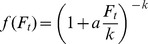
(4)where *a* is the parasitoid's searching efficiency (the proportion of total hosts encountered by parasitoids *per* unit time), and *k* is the clumping parameter of the negative binomial. *k* determines the degree of contagion resulting from parasitoid attacks: as *k* decreases, attacks become more aggregated [49]. Decreasing *k* increases the stability domain: when the host alone undergoes exponential growth, the host-parasitoid model is completely stable when 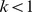 [49].

### Host density dependence

The host's probability of surviving intraspecific competition is expressed by the Maynard Smith and Slatkin [50] density dependence function *g*: 
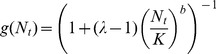
(5)


where *K* is the carrying capacity of the environment for the host population, and *b* is the coefficient determining the severity of the density dependence, *i.e.* the degree to which mortality compensates for the increase in population size. The higher the value of *b*, the greater the compensation for host competition. The dynamics of the parasitoid-free host depends on the parameter 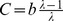. For 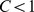, the host population is asymptotically stable (under-compensating competition); for 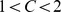, the host population reaches stability through oscillatory damping; for 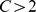, the host population is unstable (over-compensating competition). We were able to display the various host dynamics through *b* for 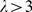, by taking discrete values not too close from the borders (*b* = 0.8, *b* = 1.5, *b* = 3.2, Table 1) which contributed to the choice of this specific function. Compared to six other models of density dependence, the Maynard Smith and Slatkin function was found to provide the best description of a wide range of data [51].

### Model analysis

We were not able to obtain analytical results from our model and we used numerical simulations to characterize the dynamics. Simulations were run with a program developed in C++ langage. Each simulation was stopped when an equilibrium (including extinction) or a periodic dynamic was reached. We considered the parasitoid population as extinct when female density decreased below 10^−6^. Initial conditions for one simulation were taken as the coexistence equilibrium values of populations in the vicinity of its position on the *a*- *λ* diagram, and simulations were run for 10 000 generations.

The dynamics of the host-parasitoid system depends mainly on which party determines the dynamics: the parasitoids (through a high host parasitism rate) or the host (through strong intraspecific competition). The relative impact of the host and of the parasitoid on the dynamics can be captured by two parameters: the host's finite rate of increase, *λ*, and the product *aK* (the expected number of hosts a single parasitoid female with a searching efficiency of *a* would parasitize when encountering a new host population with a density of *K*). However, for simplicity, we fixed *K* and represented the parasitoid dynamics as a function of the parasitoid searching efficiency only, *a*, and of the host's finite rate of increase, *λ*. The higher *a* relative to *λ*, the higher the impact of parasitism on the dynamics of the system. Conversely, at low value of *a* relative to *λ*, competition between hosts is a prime driver of the dynamics.

### The critical number of females

When the parasitoids undergo mate-finding difficulties, persistence of host-parasitoid systems may rest on the initial parasitoid density. We quantified the minimum number of female parasitoids 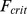 that need to be introduced to allow parasitoid establishment. This is determined numerically by introducing an increasing number of parasitoid females into a host population with a stable density equal to its carrying capacity (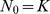). The number of hosts remaining is also of particular value for biological control: we quantified 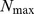
as the number of hosts at equilibrium when the population is stable, or the maximum number of hosts when the host dynamics are cyclic.

## Results

### Allee effects and risk of extinction

**Figure 1:**
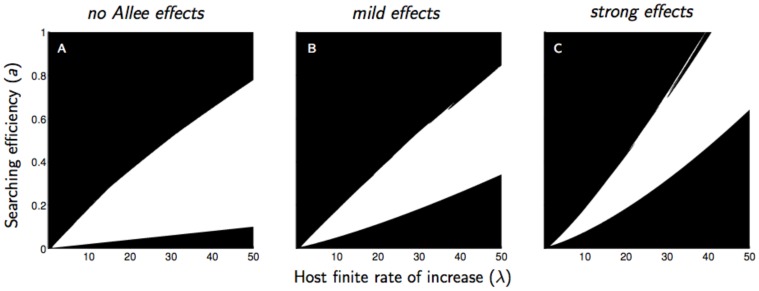
Domain of parasitoid extinction (black) and persistence (white) as a function of parasitoid searching efficiency (*a*) and host finite rate of increase (*λ*) for three intensities of a mate-finding Allee effect: no Allee effect (*α* = 0), mild effects (*α* = 5), strong effects (*α* = 20). Other assumptions are a random distribution of parasitoid attacks (*k* = 10) and a medium intraspecific competition in the host population (*b* = 1.5). Two different domains of extinction can be distinguished: the upper domain corresponding to high parasitoid efficiency and low host finite rate of increase and the lower domain, corresponding to low parasitoid efficiency and high host finite rate of increase.

When parasitoid mating success decreases with decreasing density, the domain of parameter values in which the parasitoid population goes extinct increases (figure 1). This general result appeared when we considered random attacks (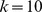) and medium compensation of the host competition (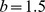). In the absence of any Allee effect, the parasitoid went extinct in two distinct domains (figure 1.a): low searching efficiencies and high host finite rates of increase (the lower domain of extinction), and high searching efficiencies and low host finite rates of increase (the upper domain of extinction). An increase of the strength of the Allee effect clearly induced an expansion of the lower domain of extinction, while the upper domain was reduced (figures 1.b, 1.c).

### Allee effects, density dependence and aggregation

**Figure 2:**
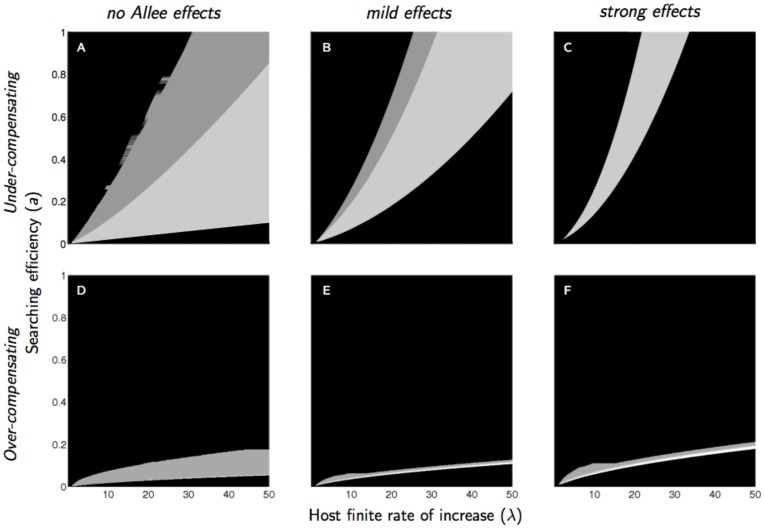
Domain of parasitoid extinction (black) and persistence (white and gray) as a function of parasitoid searching efficiency (*a*) and host finite rate of increase (*λ*) for three intensities of a mate-finding Allee effect (no Allee effect: *α* = 0; mild effect: *α* = 5; strong effect: *α* = 20) and two levels of intraspecific competition in the host population (under-compensated competition: *b* = 0.8; over-compensated competition: *b* = 3.2). A random distribution of parasitoid attacks is assumed (*k* = 10). Parasitoid persistence is underpinned by qualitatively distinct dynamics; white: asymptotic stability; light gray: damped oscillations; dark gray: cycles or chaos.

Parasitoid mating failures interacted with host density dependence to produce a complex pattern of extinction and persistence. In the absence of Allee effect, the domain of parameter values yielding parasitoid extinction was much larger for over-compensating than for under-compensating competition (figures 2.a, 2.d). The Allee effect led to an expansion of the lower domain of extinction whatever the severity of competition. However the consequences of the Allee effect on the upper domain of extinction and on the global area of parasitoid extinction were rather different depending on the competition. In a context of under-compensating host competition, the upper domain of extinction was progressively reduced, and the global area of extinction progressively increased when the Allee effect increased (figures 2.a, 2.b, 2.c). In a context of over-compensating competition, the upper domain of extinction and the global area of extinction increased when an Allee effect was introduced (figure 2.d and 2.e), but the upper domain of extinction was reduced and the global area of extinction remained equally large when the Allee effect increased from medium to strong intensities (figures 2.e, 2.f).

**Figure 3:**
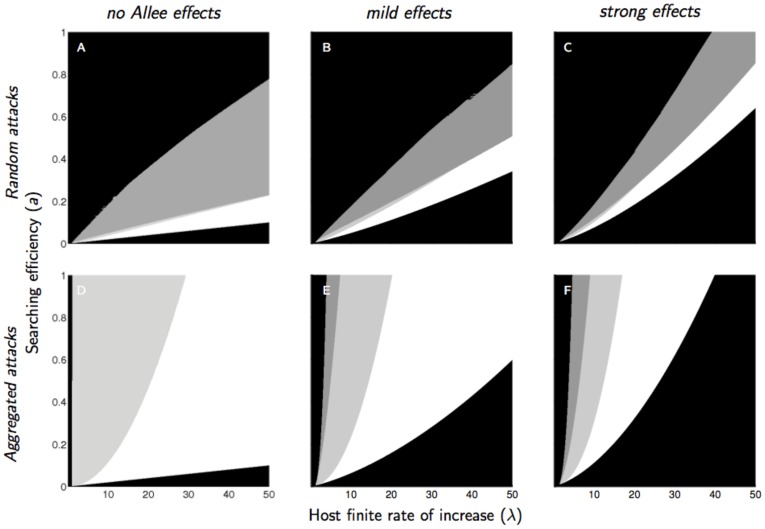
Domain of parasitoid extinction (black) and persistence (white and gray) as a function of parasitoid searching efficiency (*a*) and host finite rate of increase (*λ*) for three intensities of a mate-finding Allee effect (no Allee effect: *α* = 0; mild effect: *α* = 5; strong effect: *α* = 20) and two distributions of parasitoid attack (random: *k* = 10; aggregated: *k* = 0.8). Intraspecific competition in the host population is assumed moderate (*b* = 1.5). Parasitoid persistence is underpinned by qualitatively distinct dynamics; white: asymptotic stability; light gray: damped oscillations; dark gray: cycles or chaos.

Aggregated attacks reduced the upper domain of extinction and stabilized the persistent parasitoid in the absence of any Allee effect (figures 3.a, 3.d). The Allee effect progressively expanded both upper and lower domains of extinction with aggregated attacks (figures 3.e, 3.f).

### Qualitative dynamics and Allee effects

When the parasitoid persisted, the dynamics of the host-parasitoid system could be asymptotically stable, stable with oscillatory damping, or cyclic, from the lower domain of extinction to the upper one (figures 2 and 3).

**Figure 4:**
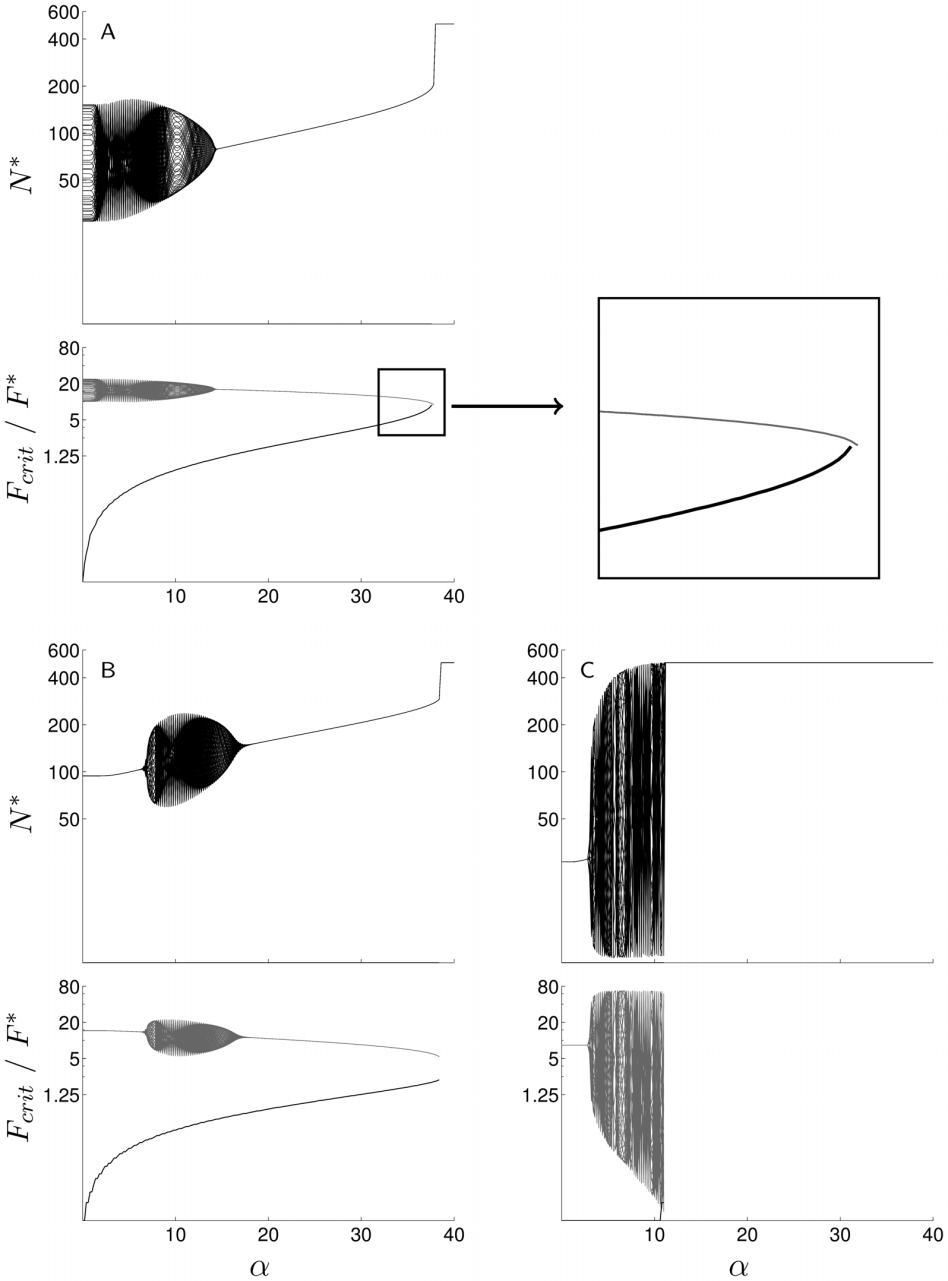
Bifurcation diagrams for hosts (black) and parasitoids (gray) populations, as a function of the intensity of a mate-finding Allee effects (*α*). Population characteristics: a) 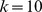, 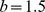, 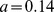, 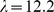; b) 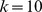, 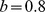, 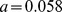, 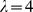; c) 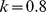, 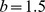, 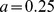, 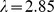. The black curve on parasitoid graphics represents 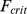.

Most cyclic parasitoid populations went extinct as soon as an Allee effect was introduced, regardless of the level of compensation of the host competition or the distribution of parasitoid attacks (figures 2 and 3). When attacks were aggregated, high parasitoid searching efficiency and low host finite rate of increase resulted in a stable equilibrium via damped oscillations (figure 3d, top left). Increasing strength of the Allee effect amplified these oscillations and produced either cyclic dynamics (if moderate) or extinctions (if strong) (figure 4c). This phenomenon was also observed when attacks occured at random, but with under-compensating competition only: persistent parasitoids in the vicinity of the upper domain of extinction could display cyclical dynamics when an Allee effect was introduced (figure 4.b).

Strong Allee effects induced a shift to higher searching efficiencies of the area corresponding to parasitoid persistence, and of all the areas corresponding to the various dynamics of persistent populations (cycles, oscillatory damping, and asymptotic stability) (figures 2.e, 2.f). This could enable a high searching efficiency parasitoid, which would not have settled without Allee effect, to become persistent (figure 4.a) and in this context Allee effects had a stabilizing impact on high searching efficiency parasitoids. For under-compensating host competition, destabilizing and stabilizing effects could appear for consecutive values of 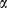, on persistent parasitoids in the vicinity of the upper domain of extinction (figure 4.b).

### Quantitative effects and biological control

The intensity of the Allee effect affected the equilibrium density of female parasitoids (F^*^). Male and female parasitoids densities at equilibrium were the highest when the population is close to the upper domain of extinction and the lowest when it was close to the lower domain of extinction. With increasing Allee effects, the population sex ratio at equilibrium became male-biased, and F^*^decreased (figures 4.a, 4.b).

**Figure 5:**
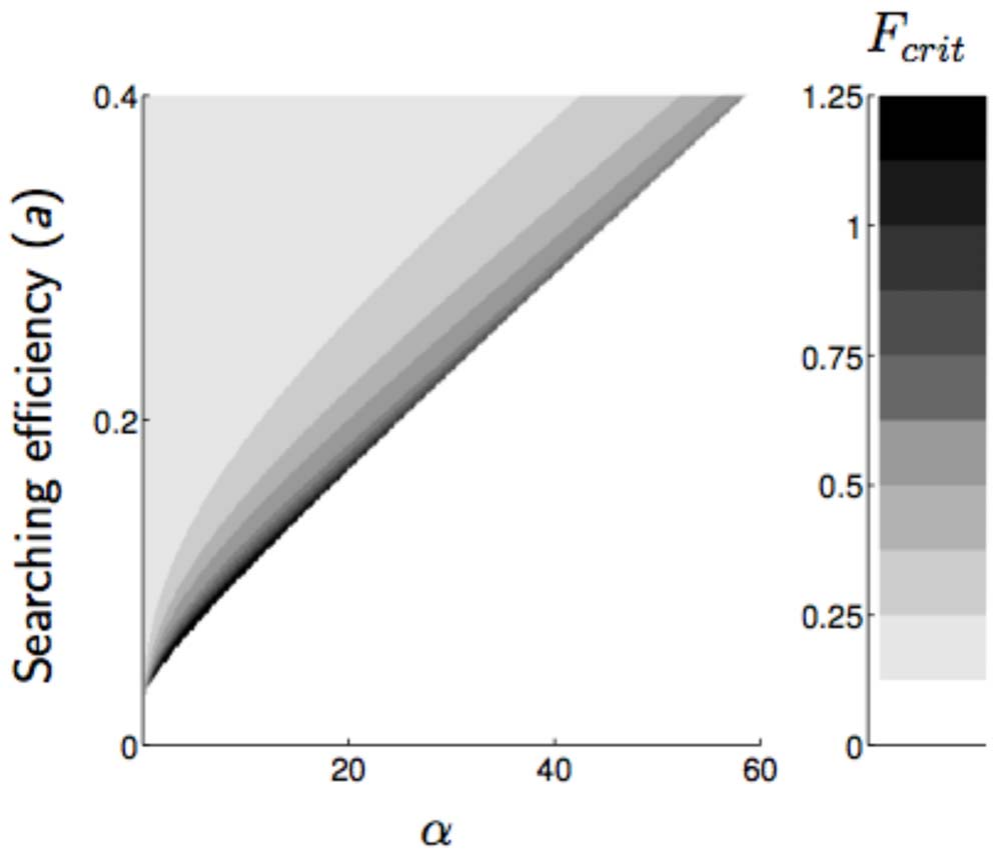
Minimum initial population density triggering parasitoid establishment (*F_crit_*) as a function of parasitoid searching efficiency (*a*) and the intensity of Allee effects (*α*). Parameter values: *b* = 1.5, 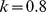, 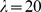, *K* = 500.

The critical number of females that have to be introduced for the parasitoid to persist (*F_crit_*) expresses the value of the extinction threshold for the parasitoid population. *F_crit_* increased with increasing Allee effect intensity (figures 4 and 5) and decreased with increasing parasitoid searching efficiency (figure 5). Compensation of the host competition did not qualitatively change this result, but *F_crit_* was quantitatively higher with over-compensating competition in the host. Over the range of parameter values studied, *F_crit_* reached its highest values for over-compensating host competition (for *α* = 20, *F_crit_* is about five times higher for *b = 3.2* than for *b = 1.5*), and its lowest values for aggregated attacks (for *α* = 20, *F_crit_* is 80% lower for aggregated than for random attacks).

The host-parasitoid system had two local equilibria when an Allee effect was introduced: host-parasitoid coexistence and parasitoid extinction. In populations with under-compensating competition, close to the lower domain of extinction, *F_crit_* increased with the Allee effect until it reached the coexistence equilibrium density: the conditions allowing the parasitoid to reach the equilibrium became more and more difficult to fulfill, and the parasitoid persistence became more and more vulnerable to a small decrease in density. Ultimately, the coexistence equilibrium became unstable and parasitoids were driven to extinction (figure 4.a). The ratio between *F_crit_* and F^*^ provides a measure of the basins of attraction of coexistence and extinction equilibria: this ratio increased with the Allee effect, wich expresses a contraction of the basin of attraction for the coexistence equilibrium and an expansion of the basin of attraction for the parasitoid extinction.

**Figure 6:**
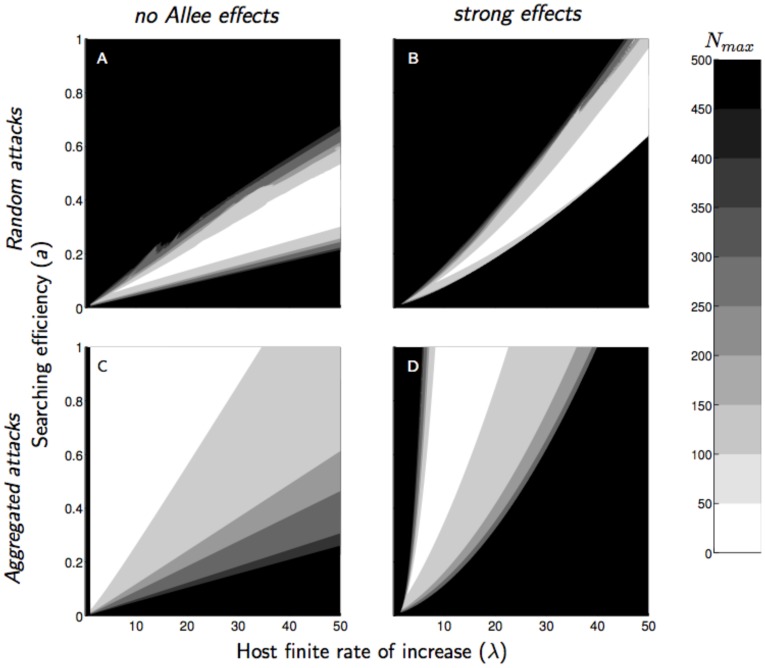
Residual host abundance after parasitism (*N*_max_) without (*α* = 0) and with (*α* = 20) a strong mate-finding Allee effect and either a random (*κ* = 10) or aggregated (*κ* = 0.8) distribution of attacks, as a function of parasitoid searching efficiency (*a*) and host finite rate of increase (*λ*). Intraspecific competition in the host population was assumed medium (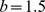)

Without Allee effect, the remaining host abundance after parasitism was naturally higher with over-compensating host competition, and reached its lowest levels - under 5% of the carrying capacity- with aggregated attacks (figure 6). Host abundance was generally increased by the Allee effect, even when it did not jeopardise the parasitoid persistence (figures 4 and 6). This increase was higher with under-compensating than with over-compensating host competition.

For the range of parameters considered in this study, an increase in 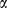 from 0 to 20 led to an increase of the host abundance between +6% and +116% (between +55% and +116% for under-compensating host competition, and between +6% and +33% for over-compensating host competition). An extreme increase in host abundance was observed for aggregated attacks and high searching efficiency parasitoids, as Allee effects lead the host to cycle at rates that could reach its carrying capacity (figures 4.c, 4.d, 6.c and 6.d).

## Discussion

Despite the major economic and ecological importance of parasitoid insects, few studies have investigated the potential consequences of parasitoid mating failures on host-parasitoid population dynamics [6]. Here we demonstrate that (1) parasitoid populations can go extinct due to Allee effects, (2) extinctions are particularly expected for parasitoids with cyclic dynamics or stable dynamics and aggregated attacks, and (3) with Allee effects, severe intraspecific competition in the host population increases parasitoid risk of extinction due to external factors decreasing population densities. We also show that parasitoid control of the host population is reduced by Allee effects, and that parasitoids producing the highest levels of control are particularly susceptible to extinction. Hence, our theoretical results show that taking mate-finding Allee effects into consideration is crucial for predicting parasitoid establishment and the consequences of Allee effects on host-parasitoid dynamics depend, to a great extent, on other characteristics of the host-parasitoid interaction.

### Parasitoid extinction and host-parasitoid dynamics with Allee effects

The Allee effect substantially increases the range of conditions prohibiting parasitoid persistence, and because it produces an extinction threshold, the Allee effect also increases the risk of parasitoid extinction by any external factor decreasing population density. An extinction threshold is commonly found in single-population models of Allee effects [8], [14], as well as in models of interacting populations [35], [36]. Our results suggest that in parasitoids, a mate-finding Allee effect at the individual level produces a strong demographic Allee effect at the population level. This implies that if the initial population size upon introduction is too small, or if once established population size decreases below a threshold, the population is doomed to extinction. The extinction threshold depends on the characteristics of both host and parasitoid populations: intrinsically stable host populations (through an under-compensating density dependence), high searching efficiency and aggregated parasitoid attacks lead to lower extinction thresholds, that is, higher resistance to external factor reducing density.

The conditions that allow parasitoids to persist and how they are modified by Allee effects are both linked to the host-parasitoid interaction. Introducing even moderate Allee effects into a host-parasitoid system with cyclical dynamics generally drives parasitoids to extinction. In the wild, very low densities should always produce mating failures, so that parasitoids with cyclical dynamics may be uncommon. Constantly, and in contrast with simple classical theoretical models, field observations suggest that cyclical parasitoid populations are rarely observed [2].

Much of our findings can be discussed from the perspective of parasitoid impact, that is, the relative influence of parasitism and intraspecific competition on the host dynamics. High-impact parasitism reflects a combination of parameters resulting in parasitoids driving most the host-parasitoid dynamics: (1) a large proportion of hosts parasitized, resulting from a high parasitoid seaching efficiency and/or a high parasitoid abundance, and (2) a moderate host intraspecific competition resulting from a high carrying capacity, and/or a low host finite rate of increase, and/or a low compensation from intraspecific competition. Host and parasitoid coexist only for relatively moderated parasitism impacts: high-impact parasitism leads to cycles and parasitoid extinction (upper domain of extinction) while low-impact parasitism leads to asymptotic extinction of the parasitoid (lower domain of extinction).

Mating failure reduces parasitism impact because it reduces the number of females produced each generation and female density at equilibrium. The “effective” parasitism impact decreases with Allee effects but the conditions for parasitism impact allowing host and parasitoid coexistence remain the same, which results in the domain of parasitoid persistence shifting toward higher searching efficiency and lower host finite rate of increase (*i.e.*, to higher parasitism impacts). For a given Allee effect intensity, the proportion of unmated females is large at low male density, a situation which occurs mainly when hosts drive the system's dynamics and parasitism impact is low. Thus, the Allee effect induces more reduction of the parasitism impact when it is already low, which in turn reduces the domain of parasitoid persistence. This mechanism shares some analogies with other processes leading to density-dependent reduction of the parasitism impact, such as parasitoid competition, autoparasitism, or host feeding on parasitized conspecifics [52], [53], [54]. While the mechanisms involved were different, these studies all pointed to a stabilizing effect on host-parasitoid dynamics. In his predator-resource model, Verdy [55] also observed stabilizing impact of the Allee effect at high predator growth rates, due to the effective reduction of the reproduction rate.

A different process may drive the parasitoid populations to extinction when attacks are aggregated or host competition is under-compensating, *i.e.*, when parasitoid populations persists despite high proportions of parasitized hosts. Without Allee effect, these populations reach stability through oscillatory damping; a mate-finding Allee effect reduces the production of female parasitoids at the bottom of the cycle (due to lower parasitoid density), which causes a release of parasitism pressure and an increase in host abundance. With a weak host competition, increased host abundance leads to increased production of female parasitoids at the top of the cycle; the existing oscillations are amplified and the parasitoid population eventually goes extinct. Severe host competition may conteract this destabilizing effect since it compensates for the increase in host abundance: the existing oscillations are not amplified and populations remain stable. This destabilizing effect is due to particular components of parasitoid growth rate (host abundance, which depends on parasitoid abundance). Similar process can also be found in predator-prey systems: destabilizing effects were indeed observed when Allee effects were introduced into the predator population in Zhou's predator-prey model [56] and in Verdy's predator-resource model [55].

Previous theoretical studies showed sex ratio variations, be they density dependent or not, affect the dynamics of haplodiploid parasitoids and may cause extinctions [45], [46], [57]. In haplodiploid populations, contrary to what occurs in diploids, mating failures induce sex ratio variations. Here, the model was developed for parasitoid wasps and we assumed haplodiploidy only. Nonetheless, comparisons between diploid and haplodiploid parasitoid systems may help to tackle the combined role of mating failures and sex ratio variations on the dynamics of host–parasitoid systems. Such comparisons are the scope of a forthcoming article.

### The intensity of Allee effects

One prime ingredient of mate-finding Allee effects is the intensity of the mate-finding difficulties, represented in our study via a single parameter, 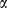. Biologically, 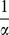 represents the mate-searching efficiency of the species [11], [24]. Various adaptations including volatile sex-pheromones and other signals generally emitted by females, as well as male cognitive and locomotory abilities, tend to increase mate-seaching efficiency; in contrast, stringent mate-choice may actually increase the time taken to find a suitable mate, and thus act as a reduction of 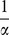 [24]. Some of the parasitoid adaptations for attracting mates (pheromone marking, reproductive aggregation) may be less efficient at low densities [24], [58], making 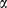 density dependent. Moreover, female parasitoids sometimes display mate choice, tending to select non-kin individuals [59]. In small groups, males are more likely to be kin, and females may choose not to mate at all if they only encounter kin males [60], [61]; in our model this would be seen as a relative increase in 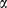.

In natural populations, the proportion of parasitoid females found to be virgin or sperm-depleted ranges from 1% to 50% [31], [39], [62], [63], [64], [65]. With the parameter values considered in this study, and the subsequent male densities at equilibrium (around 5 to 40), this proportion of virgin females is equivalent to an 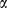 value ranging from 1 to 30.

### The framework of biological control

The ability of parasitoids to reduce host abundance, a proxy of which is the proportion of parasitized hosts, is a major criterion for biological control. Successful biological control, leaving less than 5% of the original host population, has been observed following field parasitoid introductions [4], [66]. Our model suggests that stability at this range of reduction can be obtained by considering aggregated attacks, as in the Beddington et al. model [66]. Aggregated attacks have been documented repeatedly in parasitoids, with values of *k* ranging from 0.5 to 1.6 [48], [67], [68], [69], [70]. Indeed, successful biological control has been achieved with highly aggregating species such as *Cyzenis albicans* and *Anagrus spp*.[67], [68], [71].

Our model suggests that parasitoids with aggregated attacks and a high proportion of parasitized host will be destabilized by Allee effects: a small reduction in parasitoid mate-searching ability may lead to either uncontrolled host abundances or to parasitoid extinction. If, in addition, hosts suffer strong intraspecific competition, the threshold for parasitoid extinction should be higher: parasitoids would have to be introduced at higher initial densities, and should then be more vulnerable to extrinsic factors decreasing population abundance; such factors are obviously common in agricultural landscapes.

As a conclusion, good candidates for biological control should be particularly sensitive to Allee effects. As suggested by Stiling [72], the establishment of a parasitoid population with Allee effects may depend on the characteristics of the host population. However, to fully link the results of our model to biological control, new empirical studies and meta-analyses of parasitoid introductions are necessary. If our interpretations hold true, special care with the following aspects should be taken when parasitoids are introduced: 1) parasitoid mating strategies (will their mate-searching ability be modified in a new environment?), 2) the characteristics of parasitoid dynamics and parasitoid attacks (what would be the consequences of a small decrease in the parasitoid mate-searching ability?), and 3) the nature of the host density dependence, in relation to the density of parasitoids that needs to be introduced and the environmental changes to which parasitoids may be subjected (will their density remain above the extinction threshold?).

## Supporting Information

Figure S1
**Analytical results for the stability of the one-sex host-parasitoid model.** The proportion of host abundance remaining with parasitism (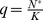) and the host growth rate (*r*) are plotted for three levels of intraspecific competition in the host population and three distribution of parasitoid attacks. From left to right: the host is stable with exponential damping (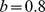), stable with asymptotic damping (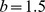), and unstable (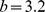); from top to bottom: random attacks (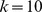), medium aggregation (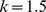), and strong aggregation (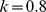). Some key values of *q* are highlighted: plain line: 0.8, dashed line: 0.5, dashed-dotted line: 0.3, dotted line: 0.05.(TIF)

Text S1 **Analytical results.**
(DOC)
