# Supplementary figures and images for: Host-Parasitoid Dynamics and the Success of Biological Control When Parasitoids Are Prone to Allee Effects
Source: PLoS One. 2013 Oct 7;8(10):e76768. doi: 10.1371/journal.pone.0076768 (PMC3792096; doi:10.1371/journal.pone.0076768)

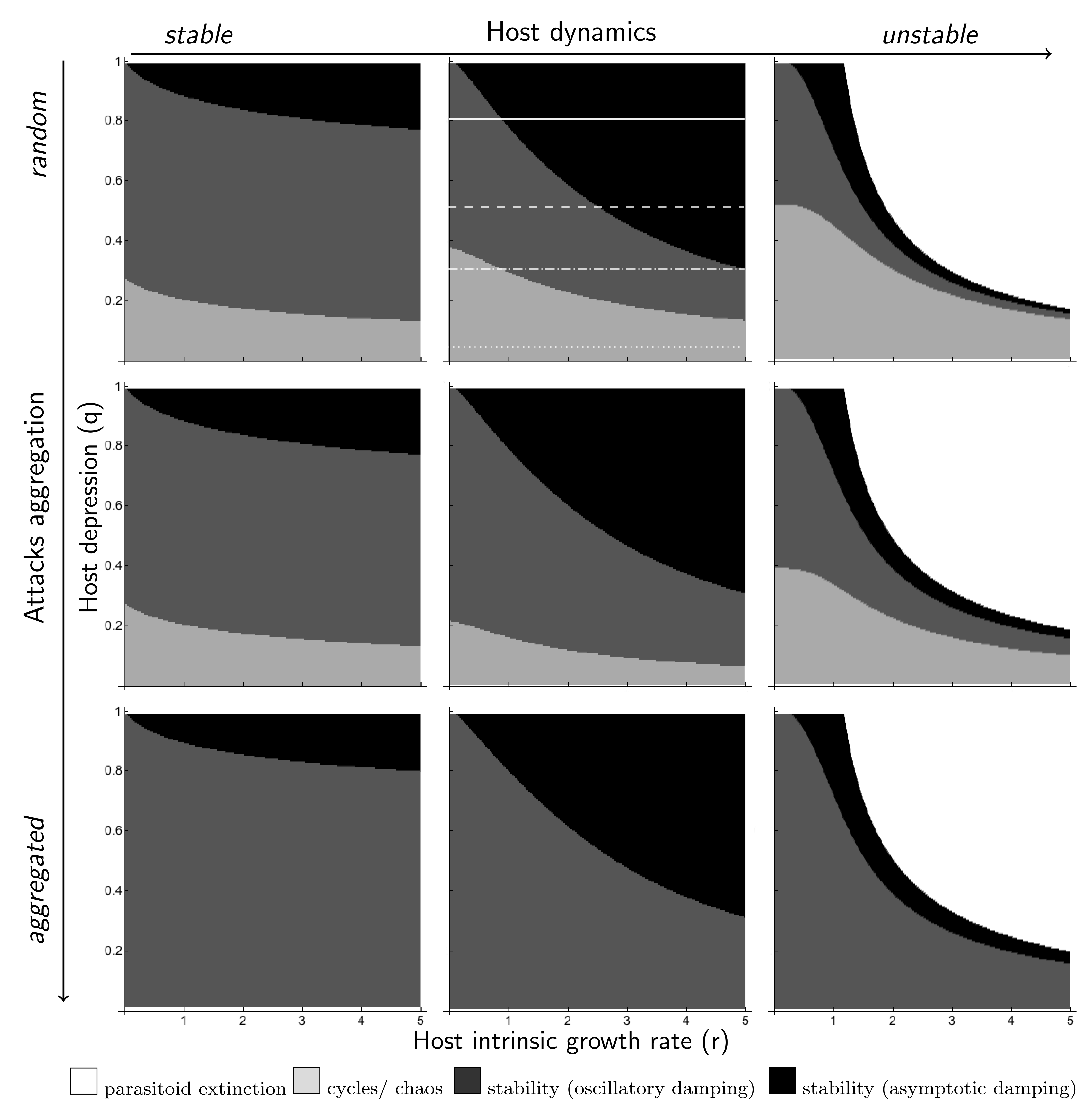

Supplement: Figure S1 — Analytical results for the stability of the one-sex host-parasitoid model. The proportion of host abundance remaining with parasitism () and the host growth rate (r) are plotted for three levels of intraspecific competition in the host population and three distribution of parasitoid attacks. From left to right: the host is stable with exponential damping (), stable with asymptotic damping (), and unstable (); from top to bottom: random attacks (), medium aggregation (), and strong aggregation (). Some key values of q are highlighted: plain line: 0.8, dashed line: 0.5, dashed-dotted line: 0.3, dotted line: 0.05. (TIF) [file pone.0076768.s001.tif]
