# Supplementary material for: Host-Parasitoid Dynamics and the Success of Biological Control When Parasitoids Are Prone to Allee Effects
Source: PLoS One. 2013 Oct 7;8(10):e76768. doi: 10.1371/journal.pone.0076768 (PMC3792096; doi:10.1371/journal.pone.0076768)
Supplement: Text S1 — Analytical results. (DOC) [file pone.0076768.s002.doc]

**Text S1. Analytical Results.**

## The one-sex model

# Conditions of stability

We defined the parameter q as the extent to which the host equilibrium ($N^{*}$) is depressed below its carrying capacity K [33].

$q=\frac{N^{*}}{K}$ (8)

This parameter decreases with the searching efficiency, *a*, as parasitoids with a high searching efficiency tend to reduce their host populations to a greater extent; it increased with *b*, $\frac{1}{K}$ and the host intrisic growth rate $r=(\lambda-1)^{\frac{1}{b}}$, as a high level of density dependence compensated for the mortality due to the parasitoid. The equilibrium can be expressed as a function of the *q* parameter:

$\left\{\begin{matrix} P^{*}=\frac{k}{a}\left(\frac{1+(\lambda-1)q^{b}}{\lambda}\right)^{^{-\frac{1}{k}-1}} \\ N^{*}=\frac{P^{*}}{d\left(1+(\lambda-1)q^{b}\right)^{-1}-\frac{d}{\lambda}} \end{matrix}\right.$ (9)

The conditions required for the stability of the model were explored through a linearized stability analysis using the method of May (1974).

The Jacobian matrix of this set of equations is as follows:

$$Jac=\left\{\begin{matrix} 1-b-\frac{b}{1+(\lambda-1)q^{b}} & -\frac{c}{d}\frac{1-\left[\lambda^{-1}\left(1+(\lambda-1)q^{b}\right)\right]^{\frac{1}{c}}}{\left(1+(\lambda-1)q^{b}\right)^{-1}-\frac{1}{\lambda}} \\ \frac{d}{\lambda}\left[1-b-\frac{b}{1+(\lambda-1)q^{b}}\right]\left[\frac{\lambda}{1+(\lambda-1)q^{b}}-1\right] & \frac{d}{\lambda}\left[\frac{c}{d}\frac{1-\left[\lambda^{-1}\left(1+(\lambda-1)q^{b}\right)\right]^{\frac{1}{c}}}{\left(1+(\lambda-1)q^{b}\right)^{-1}-\frac{1}{\lambda}}\right] \end{matrix}\right\}$$

(10)

We set $A=1-b-\frac{b}{1+(\lambda-1)q^{b}}$ and $B=-\frac{c}{d}\frac{1-\left[\lambda^{-1}\left(1+(\lambda-1)q^{b}\right)\right]^{\frac{1}{c}}}{\left(1+(\lambda-1)q^{b}\right)^{-1}-\frac{1}{\lambda}}$

Hosts and parasitoids reach a stable equilibrium if, and only if:

$A<2-\sqrt{\frac{d}{\lambda}AB\left(\frac{d}{\lambda}AB+4\lambda\left(1+(\lambda-1)q^{b}\right)^{-1}-2\right)}-\frac{d}{\lambda}B$ (11)

# Results

With b=2, the Maynard Smith density dependent function of the host gives way to a Mann Ricker function. Our model then becomes very similar to May et al. (1981)'s model [32].

Four distinct regions can be described, as the equilibrium is either asymptotically stable (black), reached by oscillatory damping (dark gray), unstable with oscillations (light gray), or corresponds to asymptotic extinction of the parasitoid (white) (supplementary figure 1). Stability is reached for intermediate values of *q*, and decreases with *r*.

For $b<1$, the competition is under-compensating and hosts alone are asymptotically stable. Thus, at low values of host depression, the host dynamics control the system, and are always asymptotically stable. For $1<b<2$, hosts become stable with oscillatory damping, and the dynamics of the model do not vary much. However, for $2<b$, the competition is over-compensating, with hosts that are unstable alone: at low host depression, they result in asymptotic extinction of the parasitoid. The more unstable the host, the less likely the parasitoid is to reach a non-null equilibrium without strong depression of the host population. The *b* parameter controls the stability of the model particularly at high values of *q* when the host population controls the dynamics.

Heterogeneity in parasitoid attacks (small values of *k*) increases the stability region at low values of *q.* For small values of *q* and of *r*, unstable oscillations give way to oscillatory damping, whereas oscillatory damping gives way to exponential damping. This finding parallels those reported in several previously published studies [32, 49]. For $k<1$, there is no unstable oscillations mode. When parasitoid attacks control the dynamics, the clamping parameter, *k,* controls the stability at low values of *q*.

The regions of action of parameters *b* and *k* do not coincide. For *b* and $k<1$, the host parasitoid system is always stable.
